# Detection of *Schistosoma* Antibodies and exploration of associated factors among local residents around Inlay Lake, Southern Shan State, Myanmar

**DOI:** 10.1186/s40249-016-0211-0

**Published:** 2017-03-01

**Authors:** Htin Zaw Soe, Cho Cho Oo, Tin Ohn Myat, Nay Soe Maung

**Affiliations:** 1 University of Community Health, Magway, Myanmar; 2 University of Medicine (Mandalay), Mandalay, Myanmar; 3 University of Medicine (I), Yangon, Myanmar; 4 University of Public Health, Yangon, Myanmar

**Keywords:** Schistosomiasis, Associated factors, Inlay Lake, Myanmar, Seroprevalence, Elisa

## Abstract

**Background:**

Schistosomiasis is a chronic parasitic disease caused by blood flukes (trematode worms) of the genus *Schistosoma*. Its transmission has been reported in 78 countries affecting at least 258 million people world-wide. It was documented that *S. japonicum* species was prevalent in Shan State, Myanmar, but the serological study was not conducted yet. General objective of the present study was to detect schistosoma antibodies and explore associated factors among local residents living around Inlay Lake, Nyaung Shwe Township, and Southern Shan State, Myanmar.

**Methods:**

An exploratory and cross-sectional analytic study was conducted among local residents (*n* = 315) in selected rural health center (RHC) areas from December 2012 through June 2013. The participants were interviewed with pretested semi-structured questionnaires and their blood samples (serum) were tested using Schistosomiasis Serology Microwell ELISA test kits (sensitivity 100% and specificity 85%) which detected IgG antibodies but could not distinguish between a new and past infection. Data collected were analysed by SPSS software 16.0 and associations of variables were determined by Chi-squared test with a significant level set at 0.05.

**Results:**

Schistosoma seroprevalence (IgG) in study area was found to be 23.8% (95% *CI*: 18.8–28.8%). The present study is the first and foremost study producing serological evidence of schistosoma infection—one of the neglected tropical diseases—in local people of Myanmar. The factors significantly associated with seropositivity were being male [*OR* = 2.6 (95% *CI*: 1.5–4.49), *P* < 0.001], residence [*OR* = 3.41 (95% CI: 1.6-7.3), *P* < 0.05 for *Khaung Daing* vs. *Min Chaung*] and education levels [*OR* = 4.5 (95% *CI*: 1.18–17.16),

*P* < 0.05 for illiterate/3Rs level vs. high/graduate and *OR* = 3.16 (95% *CI*: 1.26–7.93),

*P* < 0.05 for primary/middle level vs. high/graduate] all factors classically associated with risk of schistosoma infection. None of the behavioural factors tested were significantly associated with seropositivity.

**Conclusion:**

Schistosoma infection serologically detected was most probably present at some time in this location of Myanmar, and this should be further confirmed parasitologically and kept under surveillance. Proper trainings on diagnosis, treatment, prevention and control of schistosomiasis should be provided to the healthcare providers.

**Trial registration:**

ISRCTN ISRCTN73824458. Registered 28 September 2014, retrospectively registered.

## Background

Schistosomiasis is a chronic parasitic disease caused by blood flukes (trematode worms) of the genus *Schistosoma*. There are two major forms of schistosomiasis—intestinal and urogenital. Species (with their geographical distributions) causing intestinal forms are *Schistosoma mansoni* (Africa, the Middle East, the Caribbean, Brazil, Venezuela, Suriname), *S. japonicum* (China, Indonesia, the Philippines), *S. mekongi* (several districts of Cambodia and the Lao People’s Democratic Republic) and *S. guineensis* and related *S. intercalatum* (rain forest areas of central Africa). Species (with its geographical distributions) causing urogenital form is *S. haematobium* [Africa, the Middle East, Corsica (France)]. Schistosomiasis transmission has been reported in 78 countries among which 52 countries are endemic with moderate to high transmission, affecting at least 258 million people world-wide. The disease is prevalent in tropical and subtropical areas especially in poor communities without access to safe water and adequate sanitation [1]. Regarding impact, the disease is second only to malaria as the most devastating parasitic disease [2]. In the Mekong River basin, the first case of schistosomiasis was reported in 1957. In the 1960’s, endemic areas of the infections were discovered in Khong Island, Laos, to Kratie province, Cambodia. Their profiles were similar to those of schistosomiasis caused by *S. japonicum.* A new intermediate snail host, *Neotriculka aperta* was identified and the Mekong strain of schistosome was accepted as *S. mekongi* in 1978 [3]. A serosurvey in upper Rejang River Basin Sarawak Malaysia, the site of the multibillion Ringgit hydroelectric power project, showed 6.8% of the individuals surveyed were schistosoma seropositive determined by an Enzyme-linked Immunosorbent Assay (ELISA) method [4]. *S. japonicum* and related *S. mekongi* were found in Shan State, Burma (Myanmar) [5, 6] but confirmation methods were not described in literature. People in contact with infested water become infected when larval forms of parasites released by freshwater snails penetrate their skin. With the rise in eco-tourism and travel “off the beaten track”, increasing numbers of tourists are contracting schistosomiasis. At times, tourists present with severe acute infection and unusual problem including paralysis [1]. The Inlay Lake is situated in southern part of Shan State. Local people live on fisheries, floating agriculture and irrigated agriculture which are conducive to schistosomiasis by intensive freshwater contact. Moreover the Inlay Lake is one of the tourist attraction sites and those visiting there may contract the infection as well. Due to the facts that Myanmar is adjacent to two schistosomiasis endemic countries—China and Laos; *S. japonicum* and *S. mekongi* species occurred in Shan State, Myanmar, and Myanmar has no data on schistosomiasis, it was decided to conduct the present study on schistosomiasis among the local residents at risk living around the Inlay Lake, Southern Shan State. The general objective was to detect the schistosoma antibodies and explore associated risk factors for schistosomiasis.

## Methods

### Study design, areas, populations and period

The study design was an exploratory and cross-sectional analytic design. Study areas were rural areas near the Inlay Lake, Southern Shan State, Myanmar, approximately within 5 kilometers of the lake. Origin of water in the lake comes from nearby mountains and water from the lake flows down into *Bilu* Creek where there is a major hydroelectric power plant of Myanmar. Study populations were local residents at risk whose occupations were water-related, for example, those working at fishery, ferry boat transportation, floating and/or irrigated agriculture and both genders, aged between 18 and 60 years as inclusion criteria. Exclusion criteria were those who were ill and unwilling to participate in the study. Study period was from December, 2012 to June, 2013.

### Sample size and sampling methods

Sample size was calculated as *n* = z^2^pq / *d* ^*2*^ = (1.96)^2^ (0.5) (0.5) / (0.055)^2^ = 317. (*p* = proportion of population with schistosoma antibodies is unknown, thus it was set at 0.5). Sampling method was a convenient sampling method in selection of rural health centre (RHC) areas. Out of five RHC areas four areas of *Khaung Daing*, *Min Chaung*, *Mine Thauk* and *Ma Gyi Seik* were selected and the remaining *San Kar* RHC area was left out due to a remote area. A consecutive sampling method was used in selection of participants from four selected RHC areas.

### Data collection methods

Firstly all eligible individuals with water-related work were recruited at selected RHCs in the morning. They were explained about the objectives of the study by the Principal Investigator. Then their written informed consents were obtained. Next their blood samples were taken to detect schistosoma antibodies. The collected blood samples were temporarily stored in cold boxes at 2–8 °C. After that their socio-demographic characteristics (ie. age, gender, race, education, occupation, monthly family income, housing type, domestic water supply, latrine type and waste disposal system etc.) and behavioral characteristics (ie. wearing tall boot while working at water-related workplace, habit of walking without footwear, bathing, swimming and washing clothes in and at river/creek/ditch/pond, habit of reading literature and attending health talk on schistosomiasis and using man excreta as fertilizer etc.) and health status within last 6 months were collected in face-to-face interviews by well-trained health staff members of University of Public Health and Basic Health Staff members from local RHCs using pre-tested semi-structured questionnaires. Environments of the study villages were also observed focusing on freshwater snail existence. Snail analysis was not conducted due to lack of expert. In the evening after collection of data from about 50 to 70 participants in each day the research team went back to Nyaung Shwe Hospital where collected blood samples were stored in a freezer at −20 °C. After obtaining blood samples and required data from 320 participants within a 6 day period the research team left Nyaung Shwe Township for Yangon together with collected blood samples under a strict cold chain in a large cold box at 2–4 °C.

### Laboratory tests

Serum taken from collected blood samples was tested with Schistosomiasis Serology Microwell ELISA test kits (with sensitivity 100%, specificity 85%) [7] by two well-trained microbiologists at Microbiology Department of University of Medicine (I), Yangon. The ELISA test was only for qualitative detection of IgG antibodies to schistosoma in serum and plasma. It could not detect any specific type of schistosoma. As specificity is 85% and interpretation of borderline color would reduce specificity, only well-defined yellow colored wells on test plates were counted as positive.

### Data management

Data from questionnaires were entered into a computer and analyzed by Statistical Package for Social Science (SPSS) software version 16.0 to draw frequency tables and find out the associations of variables and their odds ratios with 95% confidence interval (*CI*). Significant level was set at 0.05.

## Results

Out of 320 blood samples, five were hemolyzed and then discarded. Among the remaining 315 samples tested, 75 were found to be positive (IgG antibodies). Therefore seroprevalence is 23.8% (95% *CI*: 18.8–28.8%). Some socio-demographic characteristics of participants are described in Table 1. Majority of participants were active age grouped (50.2%), males (50.5%), from village in *Mine Thauk* RHC area (29.5%), with primary level education (49.8%) and *Inthar* nationals (95.6%). They worked as paddy farmers (47%), vegetable farmers (24.8%), paddy and floating vegetable farmers (21.9%), casual workers (4.4%), fisherman (1.6%) and engine boat driver (0.3%). Out of these characteristics, gender, residence (RHC area) and education levels were observed statistically associated with schistosoma seropositivity among the locals (*P* < 0.05) (Table 2). Male locals were 2.6 times more likely to be schistosoma seropositive than female (*P* < 0.001). Locals from *Khaung Daing* RHC area were more prone to be seropositive in overall (*P* = 0.003). In group-wise analysis those from *Khaung Daing* area were 3.41times more likely to be seropositive than those from *Min Chaung* (*P* < 0.05). Education level of the participants was also significantly associated with seropositivity in overall (*P* = 0.036). In group-wise analysis, those with illiterate/3Rs level and those with primary/middle level were 4.5 and 3.16 times more likely to be seropositive than those with high/graduate level respectively (*P* < 0.05). With regards to other socio-demographic factors, most of the participants had a monthly family income of Kyat 50 000–100 000 (55.6%), lived in thatch roofed bamboo houses (50.8%). 37.4% of participants received their domestic water from ponds and streams. In latrine types 40.4% used non-fly proof latrines and 2.5% still defecated on ground surface. Most of them (88.3%) used proper waste disposal system especially by infiltration in the ground. In connection with behavioural characteristics they were not associated with seropositivity. 7.9% used man excreta as fertilizer for growing vegetables. There was no knowledge of schistosomiasis among the participants and no health talk on schistosomiasis had been given there. The local health personnel also did not know about the existence of schistosomiasis in their living areas. Regarding health conditions among the participants within last 6 months, possibly within their memory recall range, 87 individuals (27.6%) reported they had signs and symptoms of gastrointestinal, liver and renal diseases but could not issue their documented medical records. Out of these 87 individuals, 55 (63.2%) made complaints about gastritis. But their reported diseases were not significantly associated with seropositivity.

**Table 1:** Schistosoma serological status and socio-demogaphic characteristics of participants of the study in Southern Shan State, Myanmar, 2012–2013 (*n* = 315)

Variables	Category	Frequency (%)
Schistosoma serological status	Seropositive	75 (23.8)
	Seronegative	240 (76.2)
Age (year)	<20	15 (4.8)
	20–39	158 (50.2)
	40–59	132 (41.9)
	60	10 (3.1)
Gender	Male	159 (50.5)
	Female	156 (49.5)
Residence (RHC area)	*Khaung Daing*	63 (20.0)
	*Min Chaung*	76 (24.1)
	*Mine Thauk*	93 (29.5)
	*Ma Gyi Seik*	83 (26.3)
Education	Illiterate	10 (3.2)
	3Rs^a^	5 (1.6)
	Primary	157 (49.8)
	Middle	93 (29.5)
	High	43 (13.7)
	Graduate	7 (2.2)

^a^Reading, Writing and Arithmetic

**Table 2:** Association between socio-demographic characteristics and schistosoma serological status of participants of the study in Southern Shan State, Myanmar, 2012–2013 (*n* = 315)

Variables	Category	Seropositive	Seronegative	*OR* (95% *CI*)	*P* value
Gender	Male	51	108	2.6 (1.5–4.49)	<0.001
	Female	24	132		
Residence (RHC area)	*Khaung Daing*	26	37	3.41 (1.6–7.3)	<0.05
	*Min Chaung*	13	63	1	-
	*Mine Thauk*	18	75	1.16 (0.54–2.49)	>0.05
	*Ma Gyi Seik*	18	65	1.34 (0.61–2.94)	>0.05 0.003*
Education level	Illiterate/3Rs	5	10	4.5 (1.18–17.16)	<0.05
	Primary/Middle	65	185	3.16 (1.26–7.93)	<0.05
	High/Graduate	5	45	1	- 0.036*

*Overall significance

## Discussion

The present study is the first and foremost study producing serological evidence of schistosoma infection at some time in local people of Myanmar. The participants were those at risk and if the whole population in the study area was examined the seroprevalence would be much smaller than 23.8%. Parasitological examination on collected stool specimen was not done because of lack of some facilities in the institute. For people from non-endemic or low transmission areas, serological and immunological techniques may be useful in detecting first evidence of infection [8]. ELISA method is useful as a screening test but remains positive after drug treatment [6]. Serological test is used to diagnose the less advanced infection or light infection where egg shedding may not be consistent in travellers. The test does not distinguish between past and current infection. It is indicative of only schistosoma infection at some time [9–11]. Stool or urine examination is a gold standard tool but it requires adult worms to be producing eggs and its sensitivity is also low especially with light infection and it takes about 6 weeks for eggs to be detected after initial infection [12]. Kato-Katz thick smear method is a standard method recommended by World Health Organization (WHO) for diagnosis of intestinal schistosomiasis but immunodiagnostic testing remains the best method for diagnosing schistosoma infection in the area of low intensity of infection when sensitivity and specificity is satisfactory [13]. Therefore it is justified that ELISA test used in the present study has satisfactory sensitivity and specificity levels. In the world map of geographical distribution of schistosomiasis Myanmar is shown as a non-endemic country till 2011 [14]. This might need to be revised due to the supporting facts that *S. japonicum* and *S. mekongi* occurred in Myanmar [5, 6] and schistosoma seropositivity found in the present study. It is also said that 4 to 5 decades back schistosoma infected cases were detected in the study area by routine stool examinations among the locals. But these data could not be traced (Myo Khin, Dr, personal communication). The reason of male preponderance in the seropositive of the present study may be their more exposure to infected snail-infested water bodies in their living areas such as fishing and swimming. In some studies males are also found to be more prone to schistosoma infection. Male gender was associated with *S. haematobium* infection (*OR* = 1.81, 95% *CI*: 1.06-3.07) in a study in Malawi where 1,139 pupils from primary schools participated [15]. Similarly males presented a 3.39 time (95% *CI*: 2.2-5.3) higher risk of getting *S. mansoni* infection in a study in Brazil (*n* = 858) [16]. In another study in Brazil prevalence of schistosomiasis was higher in males than females (30% vs 18%) showing that the infection was associated with men and their freedom to move everywhere including the places like forest, mud, streams and rivers [17]. Therefore the present study is compatible with aforementioned studies in terms of male gender as a risk factor. Locals in *Khaung Daing* RHC area are more liable to be seropositive than other areas and the possible contributing factors should be sought further. In education it is also noted that the lower the education level, the greater the chance of contracting the infection suggesting that education is essential in control and prevention of diseases. These findings support the conclusion that a positive serology actually reflected true schistosoma infection.

The participants were found to be from a lower socioeconomic class. One of studies in Uganda, wealth index (scores representing the wealth of the households) was used as a risk factor for infection with *S. mansoni* after controlling for water contact and treatment with praziquantel in data analysis. The adjusted odds ratio of being infected for the lowest level of wealth index compared to the highest was 10.42 (95% *CI*: 3.38–32.36) [18], indicating that the lower the socioeconomic class, the greater the chance of contracting the infection. Other suggestions to control the schistosoma infection in the study areas are to keep local water sources safe not to be contaminated with human excreta, to provide necessary materials to build sanitary latrines, to correct the wrong habit of defecating on ground surface by giving health education and to encourage users of man excreta to replace it with alternative safe methods in growing vegetables. Cases with gastrointestinal signs and symptoms need to be urgently followed up by searching the eggs in stool and cercaria in local snails known to be potential intermediate hosts. In the study areas there were a lot of freshwater snails observed in different sizes that also need malacological surveys and analysis in future. Due to the evidence of schistosoma seropositivity found in the present study, schistosoma infection should be placed in the list of Diseases under National Surveillance in Myanmar and its prevalence must be monitored periodically. Suspected cases should be confirmed by routine stool or urine examination and promptly treated with standard drug—praziquantel (40 mg/kg body weight) single oral dose [19]. Then mass drug treatment should be considered in the area of high endemicity. Being an exploratory study there were some limitations. They were non-probability sampling methods and the results obtained were thus not representative to the whole population, seropositvity was 23.8%, thus sample size was underestimated, and parasitological examination to further confirm the cases was not carried out.

## Conclusions

Schistosoma infection had been first serologically detected by the present study among local people in Southern Shan State of Myanmar. Its magnitude in people at risk was disclosed and the disease should be further confirmed parasitologically and kept under surveillance. Healthcare providers should also be provided with proper trainings on diagnosis, treatment and prevention and control of schistosoma infection and instructed to report any clinically suspected or diagnosed cases.

## Abbreviations

ELISA: Enzyme-linked immunosorbent assay
RHC: Rural health centre
SPSS: Statistical package for social Science
3Rs: Reading, writing and arithmetic in education
