# Erratum to: Detection of *Schistosoma* Antibodies and exploration of associated factors among local residents around Inlay Lake, Southern Shan State, Myanmar

**DOI:** 10.1186/s40249-017-0334-y

**Published:** 2017-07-13

**Authors:** Htin Zaw Soe, Cho Cho Oo, Tin Ohn Myat, Nay Soe Maung

**Affiliations:** 1 University of Community Health, Magwe, Magway Myanmar; 2 University of Medicine (Mandalay), Magwe, Mandalay Myanmar; 3 University of Medicine (I), Yangon, Myanmar; 4 University of Public Health, Yangon, Myanmar

## Erratum

The information on trial registration in this article [1] was included in error.

The original article was corrected.

### Notes:

The online version of the original article can be found at 10.1186/s40249-016-0211-0.
